# A Synergetic Screening Approach with Companion Effector for Combination Therapy: Application to Retinoblastoma

**DOI:** 10.1371/journal.pone.0059156

**Published:** 2013-03-19

**Authors:** Jeni P. Mahida, Christophe Antczak, Daniel DeCarlo, Kathryn G. Champ, Jasmine H. Francis, Brian Marr, Arthur S. Polans, Daniel M. Albert, David H. Abramson, Hakim Djaballah

**Affiliations:** 1 HTS Core Facility, Molecular Pharmacology and Chemistry Program, Memorial Sloan Kettering Cancer Center, New York, New York, United States of America; 2 Ophthalmic Oncology Service, Department of Surgery, Memorial Sloan Kettering Cancer Center, New York, New York, United States of America; 3 Department of Ophthalmology and Visual Sciences, University of Wisconsin, Madison, Wisconsin, United States of America; Vanderbilt University, United States of America

## Abstract

For many cancers, the lack of potency and the toxicity of current drugs limits the dose achievable in patients and the efficacy of treatment. Among them, retinoblastoma is a rare cancer of the eye for which better chemotherapeutic options are needed. Combination therapy is a compelling approach to enhance the efficacy of current treatment, however clinical trials to test rationally designed combinations of approved drugs are slow and expensive, and limited by our lack of in-depth knowledge of drug specificity. Since many patients already turn to nutraceuticals in hopes of improving their condition, we hypothesized that certain approved drugs could potentially synergize with widely consumed supplements. Following this hypothesis, we devised an alternative screening strategy aimed at taking advantage of a bait compound such as a nutraceutical with potential therapeutic benefits but low potency, by screening chemical libraries for approved drugs that synergize with this companion effector. As a proof of concept, we sought to identify approved drugs with synergetic therapeutic effects toward retinoblastoma cells in combination with the antioxidant resveratrol, popular as a supplement. We systematically tested FDA-approved drugs and known bioactives seeking to identify such pairs, which led to uncovering only a few additive combinations; but to our surprise, we identified a class of anticancer drugs widely used in the clinic whose therapeutic effect is antagonized with resveratrol. Our observations could explain in part why some patients do not respond well to treatment. Our results validate this alternative approach, and we expect that our companion effector strategy could significantly impact both drug discovery and the nutraceutical industry.

## Introduction

Current therapeutic approaches to treat cancer are limited by toxicity and/or lack of efficacy. Most conventional cytotoxic drugs currently used in the clinic are also toxic to normal cells, thus characterized by a narrow therapeutic window that limits their use. As a way to overcome their limitations as single agents, researchers explored drug combination for cancer therapy as early as in the 1960s [1]. Some of these combinations still constitute the standard care for several cancers, such as for pediatric leukemias. Unfortunately, combining cytotoxic drugs has important drawbacks. First, the broad toxicity of those agents leads to severe side effects that limit the number of drugs to be used in combination, as well as their dose. Second, the mechanism of action of conventional chemotherapeutic agents converges on a limited number of pathways, which can be overcome by cancer cells with only a few mutations directed on genes controlling apoptosis and DNA repair. Therefore, the potential of combination therapy for cancer using conventional cytotoxic drugs is limited [2].

More recent drugs targeting oncogenic pathways in cancer cells such as kinase inhibitors are limited by the appearance of resistance, even in those patients that initially respond well to treatment, due to the existence of multiple redundant signaling pathways [3]. For this reason, the massive investment in kinase inhibitors has been met with mixed results in the clinic and there is a need for an approach that would enable targeted treatments to bypass resistance mechanisms. Since the discovery of BCR-ABL mutations conferring resistance to imatinib [4], it has become clear that focusing on a single target is not sufficient to yield sustained growth inhibition, and relapse usually occurs due to the ability of cancer cells to escape from blockage of a single essential pathway [3], [5]. This observation was confirmed again with the promising selective BRAF inhibitor vemurafenib (PLX4032); despite a strong initial response, most patients relapse after a year of treatment [6] due to the emergence of various resistance mechanisms. To overcome this limitation, a compelling approach consists in combining drugs with different molecular targets to maximize potency and minimize resistance [3], [5]. Combination therapy also provides an opportunity to identify potent combinations of already approved drugs with potentially new indications, in line with the recent initiative by the National Center for Advancing Translational Sciences (NCATS) to promote the repurposing of existing drugs. However, despite its potential, there are important limitations to the rational design of combinations of approved drugs, such as our lack of in-depth knowledge of target specificity, of target/target interactions and the difficulty of identifying potent interactions with current approaches [3]. To predict the best combinations among a very large number of possible pairs is a daunting task, and flawed in nature based on our limited knowledge of target biology, signaling networks and drug specificity. Whether the presumed target of so-called targeted agents is the only or even the main actual target is often unclear [3]. Furthermore, to identify potent combinations directly in patients through dedicated clinical trials as is the current standard practice is slow and expensive. For these reasons, an unbiased combinatorial approach to evaluate drug combinations in vitro is needed [3]. Such an effort is under way at the NCI, with the systematic screening of pair-wise combinations of small molecules currently approved as oncology drugs in the United States [3], [7]. While the outcome of this study will be of interest, whether it will actually benefit patients remains unknown. Indeed, a main limitation of this approach consists in the potential combined toxicity of two targeted anticancer agents. In spite of their name, targeted drugs such as tyrosine kinase inhibitors very often lack specificity and inhibit multiple kinases, due to their binding to the ATP pocket which is conserved among kinases. A main recommendation to limit the toxicity of this class of drugs consists in improving their selectivity [8]. Therefore, while managing the toxicity of one such drug has proved possible, it is unclear whether combining two drugs with two different targets and two different sets of associated off-targets will lead to acceptable toxicity in patients.

For these reasons, we sought an alternative approach, whose rationale is rooted in the observation that many cancer patients consume supplements as a mean to improve their condition [9], [10]. Nutraceuticals very often consist of natural products, which have historically been the main source of new drugs [11], [12]. This historical success may be explained by a higher prevalence of privileged structures more likely to produce a desirable bioactivity, and natural products are widely considered an untapped source of novel therapeutic agents [11]. Nutraceuticals are normally safe in humans and therefore provide an attractive source of companion effectors that could synergize with approved drugs in absence of added toxicity [12]. Based on these observations. we devised an alternative screening strategy seeking to take advantage of small molecules of potential therapeutic benefit, non toxic in nature, but limited by low potency such as nutraceuticals.

As a proof of concept of our approach, we sought to screen chemical libraries for approved drugs that synergize with the antioxidant trans-resveratrol (referred to as resveratrol in this manuscript) widely consumed as a supplement [9], to prevent the growth of retinoblastoma cells. Retinoblastoma is a rare cancer of the eye affecting young children for which more effective chemotherapeutic approaches are needed [13]. We were previously successful in identifying already approved drugs as candidates for drug repurposing to treat retinoblastoma (HTS) [14], [15], and we sought to take advantage of our experience to identify potent combinations to treat this rare cancer. Interestingly, the natural product resveratrol was found to have antiproliferative properties toward retinoblastoma and uveal melanoma cells and is thought to cause cell death by activation of the intrinsic apoptosis pathway [16], [17], albeit at high concentrations difficult to achieve in patients. Resveratrol has generated a great enthusiasm in the research community as well as in the public due to its presumed health benefits, including the prevention of cancer [18], [19]. Its lack of potency combined with poor bioavailability limits its use as a drug as a single agent [20], but in absence of toxicity in humans, resveratrol constitutes an excellent candidate for adjuvant therapy [21], especially for the treatment of retinoblastoma [10], [16].

In this article, we report the outcome of our efforts which consisted of screening a library of bioactive compounds and FDA-approved drugs on retinoblastoma cells to identify drugs that synergize with resveratrol. We confirm the activity of identified agonists and antagonists in dose response studies against a panel of well-established cell lines covering a wide range of cancers. We present our results and discuss their implications in the context of the identification of potent drug combinations and interactions with nutraceuticals.

## Materials and Methods

### Cell Lines and Tissue Culture

The human retinoblastoma cell line Y79 was purchased from the American Type Culture Collection (#HTB-18, ATCC, Manassas, VA). The human retinoblastoma cell line RB355 [22] originally established by Dr. Brenda Gallie (University of Toronto) was kindly provided by Dr. Michael Dyer (Saint Jude Children’s Research Hospital). RB355 and Y79 cells were grown in RPMI 1640 media with 20% (v/v) fetal bovine serum (FBS), 2 mM glutamine, 1 mM sodium pyruvate, and 4.5 g/L glucose. The human retinoblastoma cell lines NCC-RbC-60 and NCC-RbC-51, the latter derived from cervical lymph node metastasis, were obtained from Riken BioResource Center (Japan). NCC-RbC-51 cells were grown in RPMI 1640 media supplemented with 2 mM glutamine, 10% (v/v) FBS and 1 mM sodium pyruvate and NCC-RbC-60 cells were grown in RPMI 1640 media supplemented with 2 mM glutamine, 10% (v/v) FBS and 50 µM 2-mercaptoethanol. The human neuroblastoma cell lines BE(1)-N [23] and BE(2)-C (ATCC #CRL-2268) were obtained from Dr. Nai-Kong Cheung (MSKCC, NY). The A2780 human ovarian cancer cell line was obtained from Dr. David Spriggs (MSKCC, NY). The OCM290 human uveal melanoma cell line [24] was obtained from Dr. Gary Schwartz (MSKCC, NY). The human hematopoietic cancer cell line Jurkat and the human promyelocytic leukemia cell line HL-60 were obtained from Dr. Mark Frattini (MSKCC, NY). The multidrug resistant HL-60 variant cell line HL-60/RV+ overexpressing P-glycoprotein [25] was obtained from Dr. David Scheinberg (MSKCC, NY). The human triple negative breast cancer cell lines HCC70 and MDA-MB-231, the human lung adenocarcinoma epithelial cell line A549 cell line and the human pancreatic cancer cell line CWR22 were purchased from ATCC. A2780, BE(2)-C, BE(1)-N, CWR22, HCC70, HL-60/RV+, Jurkat and OCM290 cells were grown in RPMI 1640 media supplemented with 2 mM glutamine and 10% (v/v) FBS. HL-60 cells were grown in IMDM media containing 10% (v/v) FBS. MDA-MB-231 cells were grown in DME high glucose/F12 media supplemented with non-essential amino acids, 10% (v/v) FBS. A549 cells were grown in F12K media supplemented with 10% (v/v) FBS. All cell lines were grown in presence of 100 units/mL penicillin and 100 µg/mL streptomycin and under atmosphere of 5% CO_2_/95% air at 37°C.

### Materials and Reagents

RPMI 1640 media, IMDM media, F12K media, glutamine, sodium pyruvate, penicillin, streptomycin, 2-mercaptoethanol, dimethylsulfoxide, glucose, phosphate-buffered saline (PBS) without Mg^+2^, Ca^+2^, 0.25% trypsin/EDTA, rhodamine phalloidin and Hoechst 33342 were purchased from Life Technologies (Carlsbad, CA). DME high glucose/F12 media was purchased from the Media Core Facility at MSKCC. Alamar Blue is a custom made solution of resazurin in PBS (0.125 mg/mL). Resazurin, trans-resveratrol (referred to as resveratrol in this manuscript) and Triton X-100 were purchased from Sigma-Aldrich (St Louis, MO). Paraformaldehyde (PFA) was obtained as a 32% (v/v) aqueous solution (# 15714-S, Electron Microscopy Sciences, Hatfield, PA). The aqueous solubility of resveratrol was assessed in UV-transparent 384 well microtiter plates (#781801, Greiner Bio-One, Monroe, NC). The cell-based assays were performed in 384-well microtiter optical imaging plates, with black clear bottom and tissue culture treated. (#3985, Corning Life Sciences, NY). The “killer mix” used as a low control in viability assays consists of a proprietary mixture of 6 cytotoxic compounds.

### Assay Development

To assess the growth kinetics of Y79 and RB355 cells, cell suspensions were dispensed into 384-well microtiter plates at cell densities ranging from 0, 1,000, 2,000, 4,000, 8,000, 16,000 to 32,000 cells per well in 45 µL growth media using Multidrop 384 liquid dispenser (Thermo Scientific, Waltham, MA) and incubated in Cytomat automated temperature- and humidity-controlled incubator (Thermo Scientific). At 24, 48, 72 and 96 h post-seeding, 5 µL Alamar Blue solution was dispensed into the assay plates using Flexdrop IV liquid dispenser (PerkinElmer, Waltham, MA) and further incubated at 37°C. After 24 h incubation, the resulting fluorescence intensity was read on LEADseeker Multimodality Imaging System (GE Healthcare, Piscataway, NJ) equipped with Cy3 excitation and emission filters and a FLINT epi-mirror.

The aqueous solubility limit of resveratrol was assessed using a NEPHELOstar microplate laser nephelometer (BMG LABTECH GmbH, Ortenberg, Germany). The turbidity of wells containing 45 µL PBS was measured post-addition of 5 µL resveratrol dilutions in 1% DMSO (v/v) final concentration, to mimic screen conditions. 24 serial doubling dilutions of resveratrol were prepared in a source plate (#AB-0781, Thermo Scientific), and 5 µL of each dilution was transferred to the assay plates using a PP-384-M Personal Pipettor (Apricot Designs, Monrovia, CA) to reach 5 mM final concentration as the upper limit. The experiment was performed in triplicate and controls consisted of standards of 0.1, 1, 10, 20, 100, 200, 800 and 1,000 Nephelometric Turbidity Units and PBS as the control buffer. The resveratrol solubility limit was defined as the resveratrol concentration yielding turbidity values greater than the average turbidity value of control buffer +3 standard deviations.

We assessed the dose response of resveratrol toward Y79 and RB355 cells in the previously optimized assay conditions of 4,000 cells seeded per well and 120 h incubation. 22 serial doubling dilutions of resveratrol were prepared in a source plate, and 5 µL of each dilution was transferred to the assay plates as previously described, to reach 400 µM in 1% DMSO as the final upper limit concentration. Y79 or RB355 cells were dispensed in 45 µL of media using Multidrop. After 96 h, 5 µL Alamar Blue solution was added to the cells and Alamar Blue fluorescence intensity was read on LEADseeker after 24 h incubation.

### Assay Control Run

To assess the robustness of the optimized assay in presence and absence of resveratrol, a control run was performed consisting of three 384-well microplates with 1,152 wells containing 1% DMSO (v/v) as the high control plates and three 384-well microplates with 1,152 wells of 1 µM ‘killer mix’ in 1% DMSO (v/v) as the low control plates. 5 µL control at 10X concentration were preplated using Personal Pipettor. Y79 and RB355 cells were dispensed in 45 µL media along with 0 or 80 µM resveratrol using Multidrop at the optimized cell seeding density of 4,000 cells per well and incubated in Cytomat at 37°C and 5% CO_2_ for 96 h. 5 µL Alamar Blue solution was added to the plates using Flexdrop and Alamar Blue fluorescence intensity was read on LEADseeker after 24 h incubation in Cytomat.

### Screen against a Library of 6,912 Compounds

The screen of a chemical library of 6,912 compounds was performed in duplicate at a compound final concentration of 10 µM in 1% DMSO (v/v). The assay was conducted according to the optimized assay conditions described above, in presence or absence of 80 µM resveratrol. Controls consisted of 1% DMSO (v/v) (high control) and 1 µM ‘killer mix’ in 1% DMSO (v/v) (low control) final concentration, in presence or absence of 80 µM resveratrol. Compound-induced reduction in viability was expressed as percentage inhibition compared to high and low controls, as defined by: % inhibition (%I) = (high control average – read value)/(high control average – low control average)×100.

Chemical screening data were loaded onto ORIS (Oncology Research Information System), a custom built suite of modules for compound registration, plating, and data management powered by ChemAxon.

### Chemical Libraries

The screened library combines 6,912 chemicals obtained from MicroSource, Prestwick, Tocris, Sigma-Aldrich and other commercial sources as previously described [26], [27]. The MicroSource Library contains 2,000 biologically active and structurally diverse compounds from known drugs, experimental bioactives, and pure natural products. The Prestwick Chemical Library is a unique collection of 1,119 bioactive chemical compounds, all off patent and selected for structural diversity and broad spectrum. Approximately 90% of the library consists of marketed drugs and 10% of bioactive alkaloids or related substances. The Tocris collection includes 1,280 high purity compounds active toward GPCRs, kinases, ion channels, nuclear receptors and transporters. The LOPAC 1280 library from Sigma-Aldrich consists of 1,280 well-characterized, high-purity compounds representing all major target classes.

### Dose Response Studies

Positives selected from the pilot screen were resupplied from vendors and dose response studies were performed toward Y79 and RB355 cells using 12 doubling dilutions from 1, 10 and 100 µM as upper limit in 1% DMSO (v/v) (final concentration) in absence of resveratrol, or with 30 or 80 µM resveratrol co-treatment using the same assay workflow as previously described. Two readouts were used for dose response studies: Alamar Blue-based viability for all cell lines and nuclei count-based proliferation for adherent cell lines. The induced percentage inhibition in each readout was calculated based on high and low controls present in the same plate treated with the same resveratrol concentration as described above. Dose response curves were fitted and maximum inhibitory concentration IC_50_ values were calculated in ORIS. For publication purposes, dose response curves were fitted using logistic four-parameter sigmoid regression equations using Sigmaplot (Systat Software Inc.). Dose response curves were deemed partial if the maximum percentage inhibition was less than 65% or if they failed to reach an asymptote at the highest concentrations tested, and calculated IC_50_ values for these curves were flagged in provided tables with an asterisk. The IC50 value for partial dose response curves with a maximal percentage inhibition lower than 50% even at the maximal tested concentration of 100 µM was reported as greater than 100 µM, since an accurate IC50 value could not be calculated.

Activity profiling of confirmed positives was performed in dose response studies toward a panel of 13 cancer cell lines using the following cell seeding densities: 500 cells per well for OCM290, 1,000 cells per well for A2780, BE(2)-C, CWR22, HCC70 and MDA-MB-231, 2,500 cells per well for Jurkat, 3,000 cells per well for HL-60 and HL-60/RV+, 4,000 cells per well for NCC-RbC-51 and NCC-RbC-60, 7,000 cells per well for BE(1)-N. The assay was performed according to the workflow previously described.

### Fixing, Nuclei Staining and Actin Staining

Adherent cells were fixed and stained for image acquisition after the Alamar Blue readout on LEADseeker according to the following protocol. Media was aspirated using an automated plate washer ELx405 (Biotek Instruments) and 50 µL of 4% PFA (v/v) in PBS was added using Multidrop and incubated for 20 min. After aspiration cells were stained with 1 µM Hoechst in PBS containing 0.05% Triton X-100 (v/v) for 20 min in the dark. The Hoechst solution was aspirated and cells were washed once with PBS, resuspended in 50 µL PBS and plates were sealed. Prior to actin staining, PBS was aspirated and cells were incubated with 10% FBS in PBS for 90 min. Following aspiration, cells were incubated with 40 µL 1∶200 dilution of rhodamine phalloidin for 30 min. Cells were washed with PBS, resuspended in 50 µL PBS and plates were sealed.

### Image Acquisition

Image acquisition was conducted on the INCA2000 (GE Healthcare). The INCA2000 is a wide-field automated epifluorescence microscope equipped with a large-chip CCD camera allowing for whole well imaging. For the nuclei count readout, images of Hoechst-stained nuclei at 4X objective magnification (0.20NA) in the DAPI channel were acquired at 350/50 nm excitation and 455/50 nm emission with an exposure time of 100 ms. One tile per well was imaged covering 100% of the well surface. For morphology studies, images were acquired at 20X magnification (0.45NA) on the INCA2000 in the blue channel for Hoechst-stained nuclei at 350/50 nm excitation and 455/50 nm emission with an exposure time of 100 ms, and in the red channel for rhodamine-phalloidin 543/22 nm excitation and 624/40 nm emission and with an exposure time of 200 ms. Four images were collected per well, each covering approximately 15–20% of the well.

### Image Analysis

For the nuclei count readout, images acquired using the INCA2000 were analyzed using object-based segmentation using a customized image analysis protocol in Developer Toolbox 1.9 software (GE Healthcare, Piscataway, NJ). Automated image analysis consisting of object based segmentation and clump breaking yielded nuclei count that was used for the quantification of cell proliferation.

## Results

### Optimization of a Viability-based Assay for the Identification of Compounds Synergizing with Resveratrol

**Figure 1:**
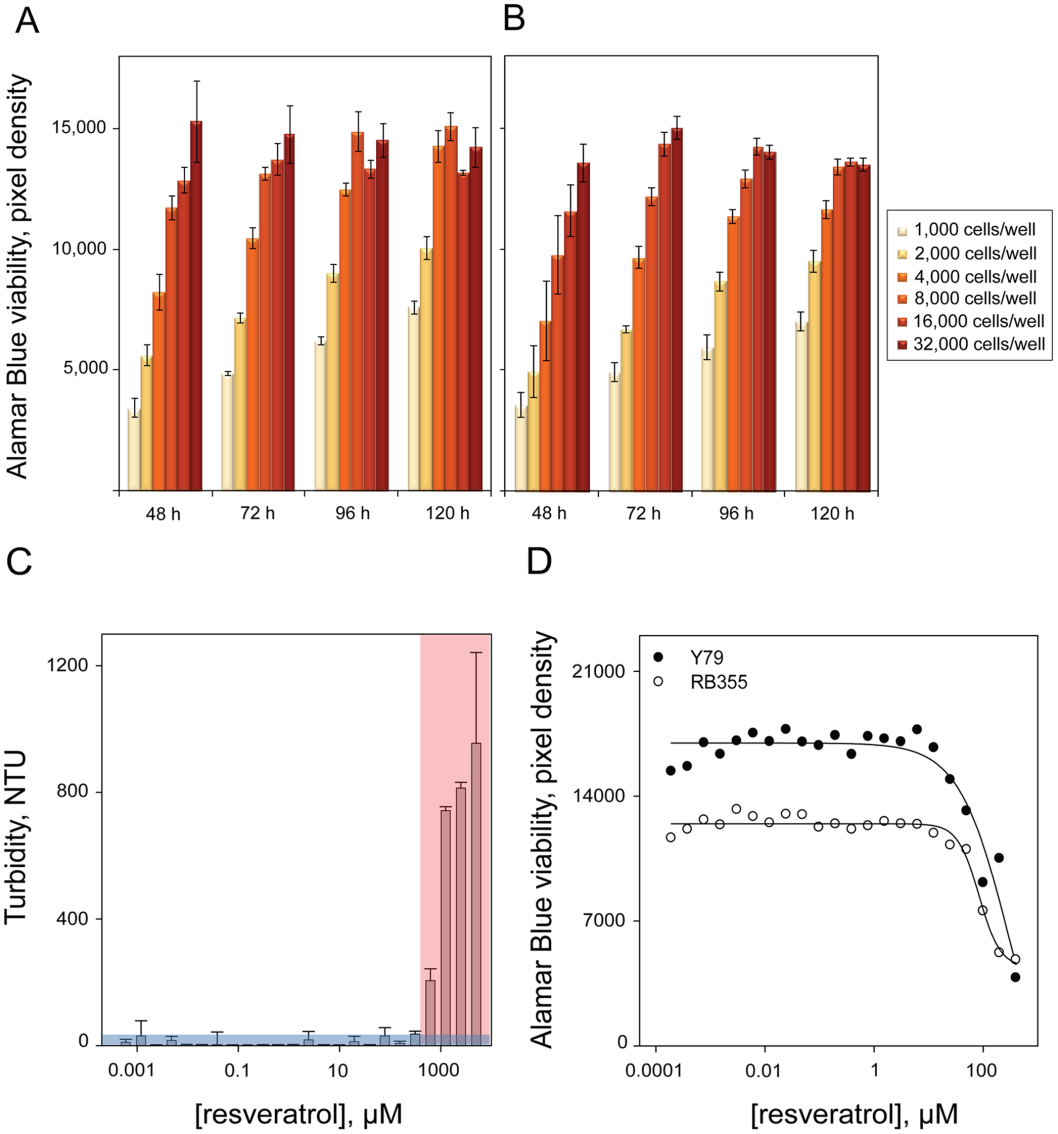
Optimization of a viability-based assay for the identification of compounds synergizing with resveratrol. Optimization of an Alamar Blue-based viability assay with the retinoblastoma cell lines Y79 and RB355 in 384-well format. Bar graph summarizing the growth kinetics of **A)** Y79 and **B)** RB355 cells over a time course of 120 hours and identifying 4,000 cells per well as the optimal cell seeding density for both cell lines. **C)** Assessment of the aqueous solubility limit of resveratrol using laser nephelometry, defined as 400 µM based on a threshold consisting of the average turbidity value of control buffer +3 standard deviations (blue). Calibration curve for turbidity standards. **D)** Dose response curve of resveratrol toward Y79 and RB355 cells using previously optimized assay conditions. Resveratrol induces approximately 50% reduction in viability for both cell lines at 80 µM and this concentration is therefore selected as the optimal resveratrol concentration for screening.

We sought to optimize an Alamar Blue-based viability assay amenable to high throughput screening (HTS) that would allow the identification of combinations with synergetic effect toward retinoblastoma cells in presence of resveratrol. For this purpose, we assessed the growth kinetics of the two retinoblastoma cell lines to be used for screening: Y79 and RB355 [14], so as to determine the optimal combination of cell seeding density and incubation time in 384-well format leading to linear growth as measured by Alamar Blue conversion [28]; this is essential to ensure accurate detection of compounds preventing cell growth during screening. Cells were seeded at densities ranging from 1,000 to 32,000 cells per well and incubated in the conditions of screening for up to 120 h. We observed a linear increase in Alamar Blue conversion up to 120 h incubation for 2,000 and 4,000 cells seeded per well, for both cell lines (**Figs. 1A & 1B**). Since seeding 4,000 cells per well and for 120 h incubation allowed for a wider signal window, those conditions were selected as optimal for the assay for both cell lines.

To ensure that the results of our study would be relevant to earlier findings, we confirmed the chemical structure of our commercial supply of trans-resveratrol by mass spectrum and NMR analysis. An important factor that we considered when designing this assay was the aqueous solubility limit of resveratrol. Because of its low potency, resveratrol must be used at high concentrations, and it is therefore essential to determine the range of concentrations where resveratrol is soluble in aqueous media. For this purpose, we used laser nephelometry in 384-well format to assess the solubility limit of resveratrol across 24 doubling dilutions from 5 mM as the maximal final concentration. Nephelometric turbidity standards were used to establish a standard curve and control buffer was used to assess the turbidity baseline of an average of about 4 Nephelometric Turbidity Units (NTUs) (**Fig. 1C**). We used a threshold of 20 NTUs consisting of the average baseline plus 3 standard deviations to define the solubility limit of resveratrol, measured as 400 µM in this experiment (**Fig. 1C**).

To determine the optimal concentration of resveratrol for screening, we assessed the dose response of resveratrol toward Y79 and RB355 in 384 well format using the previously optimized assay conditions. The aqueous solubility limit of resveratrol of 400 µM previously defined was used as the maximal final concentration of the 22 doubling dilutions to be tested in the assay. Cells were seeded at the optimal density of 4,000 cells per well and Alamar Blue conversion was read 24 h post-addition, after a total of 120 h incubation. We observed 50% reduction in signal occurred for cells treated with 80 µM resveratrol for both cell lines, and this concentration was therefore selected as the optimal resveratrol concentration for screening, since it induced a significant decrease in the viability of retinoblastoma cells while still yielding a good signal window for readout (**Fig. 1D**).

### Evaluation of Assay Robustness in the Conditions of Screening

**Figure 2:**
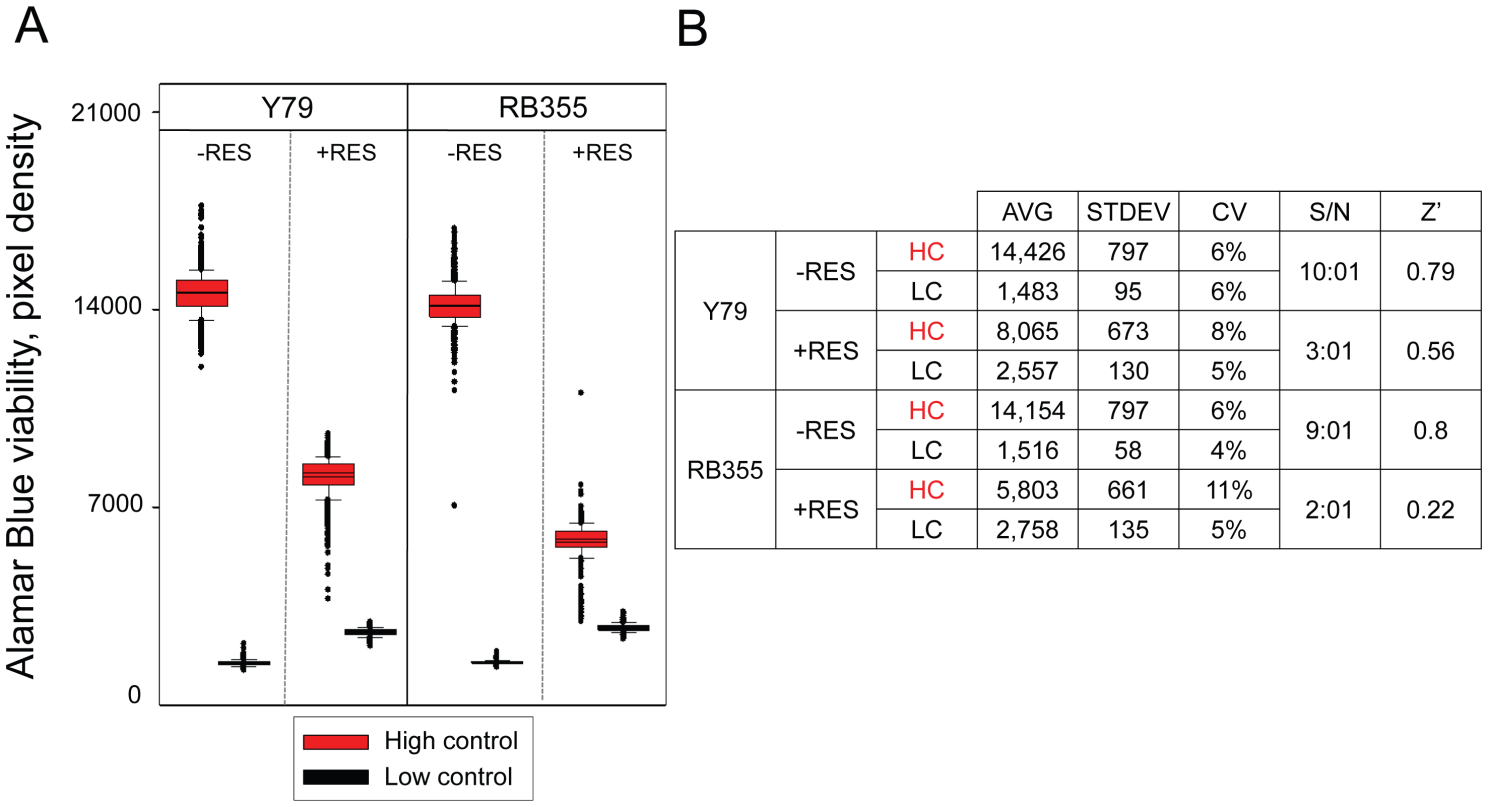
Control run experiment to evaluate the robustness of the optimized assay in the conditions of screening. A) Box plot analysis of high control and low control data toward Y79 and RB355 cells in absence (-RES) and presence (+RES) of 80 µM resveratrol. Controls consisted of 1% DMSO (v/v) (high control) and 1 µM ‘killer mix’ in 1% DMSO (v/v) (low control) as final concentration. **B)** Summary table of statistics for the assay control run. The average pixel density (AVG), standard deviation (STDEV), coefficient of variation (CV), signal-to-noise ratio (S/N) and calculated Z’ values are presented.

Evaluating the robustness of our assay for a large number of data points in the conditions of screening is essential to ensure accurate positive selection. For this purpose, we performed a control run mimicking screening conditions with Y79 and RB355 cells consisting of 1,152 high control wells containing 1% DMSO (v/v) and 1,152 low control wells containing 1 µM ‘killer mix’ in 1% DMSO (v/v) in presence and absence of 80 µM resveratrol. For Y79 cells in absence of resveratrol, we observed an average signal value of 14,426 for the high controls and 1,483 for low controls, which translated into a signal to noise ratio of 10∶1 and a calculated Z’ value of 0.79, indicative of excellent robustness. In presence of resveratrol, an expected lower average value of 8,065 was observed for high controls and 2,557 for low controls, leading to a signal to noise ratio of 3∶1 and a calculated z’ value of 0.56, still indicative of good robustness since being greater than 0.5 [29] (**Fig. 2A & 2B**). For RB355 cells in absence of resveratrol, the average value for high controls was 14,154 and 1,516 for low controls, which translated into a signal to noise ratio of 10∶1 and a calculated Z’ value of 0.8, while in presence of resveratrol, the average signal value was 5,803 for high controls and 2,758 for low controls, which translated into a signal to noise ratio of 3∶1 and a Z’ value of 0.22, indicative of poorer separation between controls (**Fig. 2A & 2B**). Our results demonstrate the good robustness of the assay for screening Y79 cells in presence and in absence of resveratrol and RB355 cells in absence of resveratrol, while the assay for screening RB355 cells in presence of resveratrol suffered from a narrower signal window. We took into account this important observation for positive selection as described below.

### Screen of a Chemical Library of 6,912 Compounds for Synergetic Resveratrol Combinations

**Figure 3:**
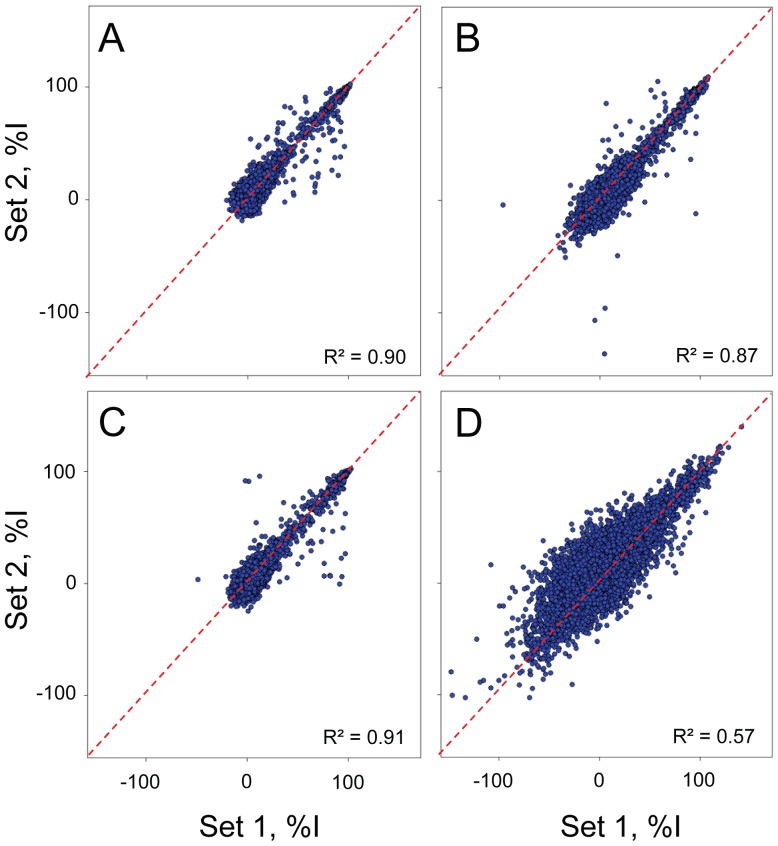
Assessment of screen reproducibility. Scatter plot analysis of four screens against 6,912 compounds performed in duplicate. The percentage inhibition (%I) for each compound and for each set of data is plotted as a scatter plot for **A)** Y79 in absence of resveratrol **B)** Y79 in presence of 80 µM resveratrol **C)** RB355 in absence of resveratrol and **D)** RB355 in presence of 80 µM resveratrol.

We screened a combined library of 6,912 FDA-approved compounds and known bioactives at 10 µM in 1% DMSO (v/v) and in duplicate using the previously optimized assay conditions. We screened both Y79 and RB355 cells, in presence or in absence of 80 µM resveratrol. To assess the reproducibility of each screen, we plotted the percentage inhibition induced by each compound in each duplicate set of data as a scatterplot (**Fig. 3**). As expected, both Y79 screens (**Fig. 3A, 3B** **)** and the RB355 screen in absence of resveratrol (**Fig. 3C**) were characterized by good reproducibility, as demonstrated by R^2^ values between 0.87 and 0.91. Not surprisingly, the RB355 screen in presence of resveratrol was noisier, with an R^2^ value of 0.57 (**Fig. 3D**), likely due to the smaller signal window in those conditions (**Fig. 2A & 2B**).

**Figure 4:**
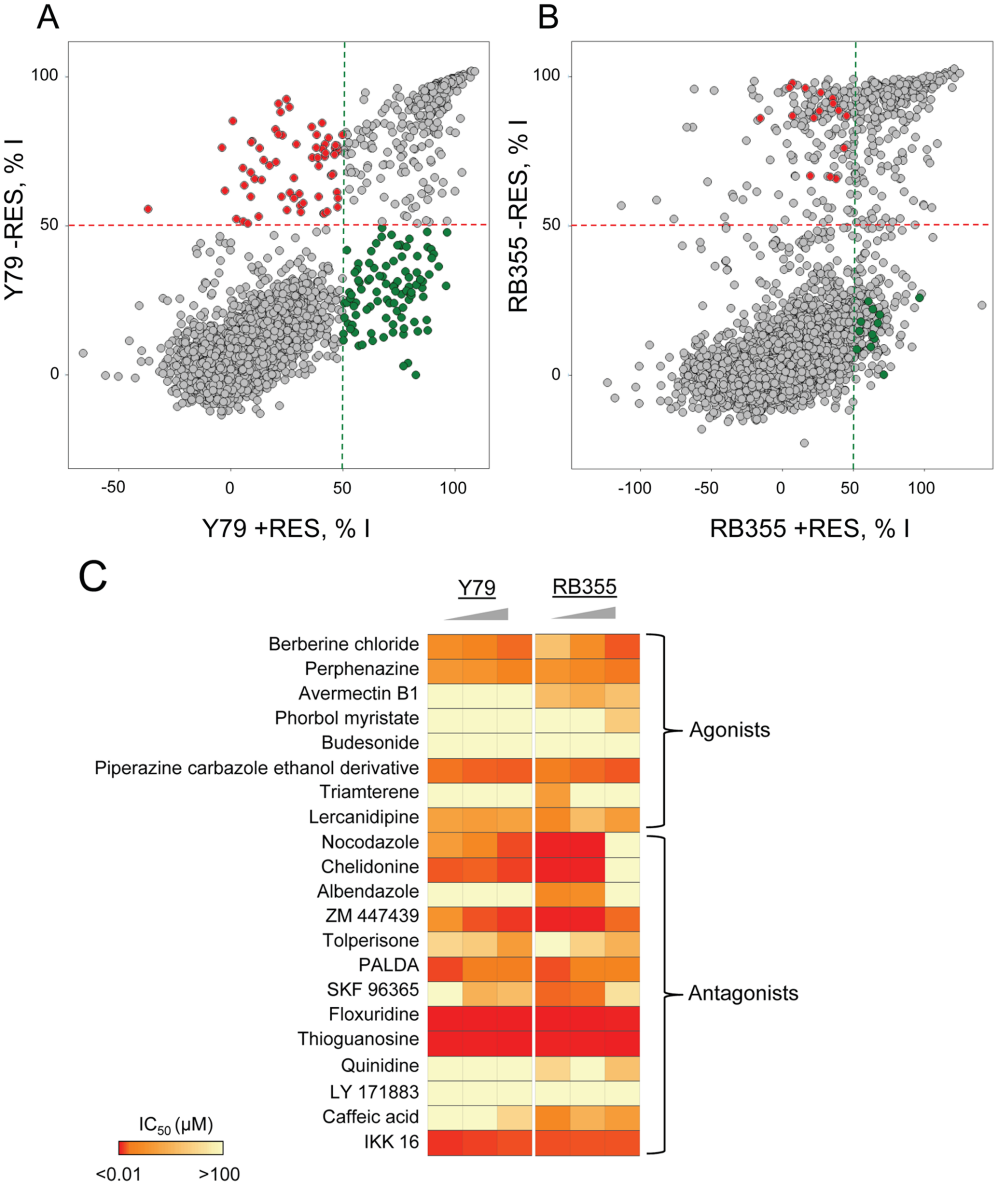
Selection and confirmation of positives. A) Scatter plot analysis of the average percentage inhibition (%I) of each compound toward Y79 cells, in absence of resveratrol (−RES) or with 80 µM resveratrol co-treatment (+RES). Initial positives with agonistic activity are selected as those compounds inducing greater than or equal to 50% inhibition in presence of resveratrol and inducing less than or equal to 50% inhibition in absence of resveratrol (green). Initial positives with antagonistic activity are selected as those compounds inducing less than or equal to 50% inhibition in presence of resveratrol and inducing greater than or equal to 50% inhibition in absence of resveratrol (red). **B)** Scatter plot analysis of the average percentage inhibition (%I) of each compound toward RB355 cells, in absence of resveratrol (−RES) or with 80 µM resveratrol co-treatment (+RES). Positives from the Y79 screen with agonistic activity in both screens are selected as those compounds inducing greater than or equal to 50% inhibition in presence of resveratrol and inducing less than or equal to 50% inhibition in absence of resveratrol (green). Positives from the Y79 screen with antagonistic activity in both screens are selected as those compounds inducing less than or equal to 50% inhibition in presence of resveratrol and inducing greater than or equal to 50% inhibition in absence of resveratrol (red). **C)** Heatmap representation of the IC_50_ values of the selected 21 positives (8 agonists and 13 antagonists) toward Y79 and RB355 cells in absence of resveratrol or with 30 or 80 µM resveratrol co-treatment. The triangle symbolizes the increasing concentration in resveratrol.

For positive selection, we took advantage of the larger signal window and better reproducibility of the Y79 screen to select initial positives with better accuracy. We plotted the performance of each compound in the Y79 screen in presence and absence of resveratrol (**Fig. 4A**). As expected, we identified several compound populations; most compounds had little effect in presence or absence of resveratrol, and some compounds were potent regardless of resveratrol co-treatment (**Fig. 4A**). Interestingly, a population of compounds was more potent in presence of resveratrol, acting as potential agonists, and perhaps surprisingly another compound population was less potent when co-treated with resveratrol, acting as antagonists (**Fig. 4A**). Based on this observation, we selected as initial agonists the 88 compounds inducing greater or equal to 50% inhibition in presence of resveratrol and less or equal to 50% inhibition in absence of resveratrol; we also selected as initial antagonists the 67 compounds inducing less or equal to 50% inhibition in presence of resveratrol and greater or equal to 50% inhibition in the absence of resveratrol (**Fig. 4A**). While this initial positive selection is solely based on screening Y79 cells, we sought to identify potential agonists and antagonists that are not cell line specific, and for this purpose we refined our positive selective based on their performance in the RB355 screen. For further studies, we selected the 12 out of 88 initial agonists (11 unique) inducing greater or equal to 50% inhibition in presence of resveratrol in the RB355 screen and less than or equal to 50% inhibition in absence of resveratrol; similarly we selected the 17 out of 67 initial antagonists (14 unique) that induced less or equal to 50% inhibition in the RB355 screen in presence of resveratrol and greater than or equal to 50% inhibition in absence of resveratrol (**Fig. 4B**). Of note, the identification of multiple copies of the same compounds provided by different suppliers as inducing the same effect is indicative of the good reproducibility of both screens.

### Confirmation of Selected Agonists and Antagonists in Dose Response Studies

**Figure 5:**
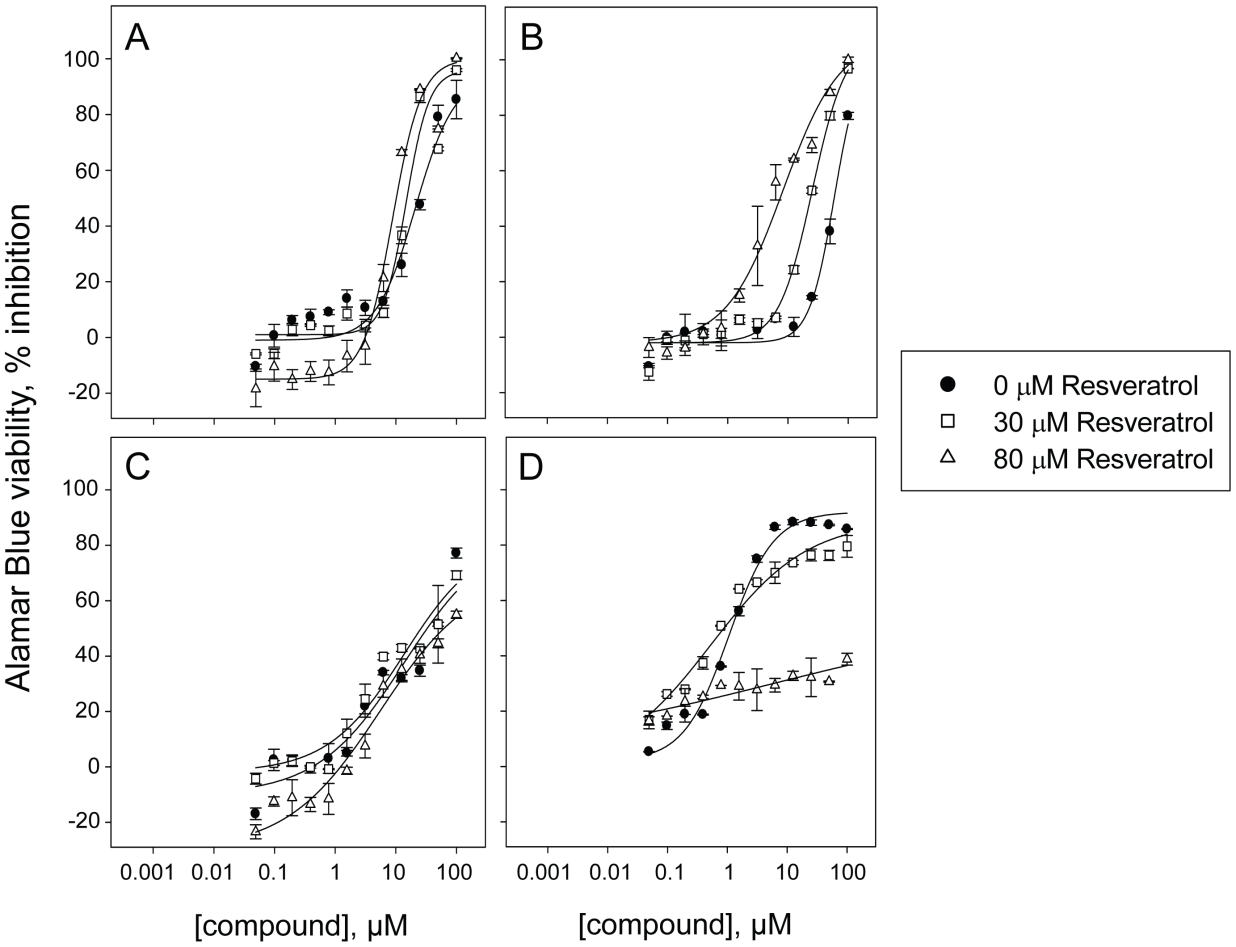
Dose response curves for confirmation of selected positives. Potency assessment of confirmed positives toward Y79 and RB355 cells in absence of resveratrol or with 30 or 80 µM resveratrol co-treatment in an Alamar Blue-based viability assay. Dose response curves of the confirmed agonist berberine chloride toward **A)** Y79 and **B)** RB355 cells inducing inhibition of cell viability with IC_50_ values of 24, 15 and 8.6 µM toward Y79 and 59, 23 and 6.8 µM toward RB355 cells in absence of resveratrol or with 30 or 80 µM resveratrol co-treatment. Dose response curves of the confirmed antagonist nocodazole toward **C)** Y79 and **D)** RB355 cells inducing inhibition of cell viability with IC_50_ values of 34, 21 and 5.6 µM toward Y79 and 1.1, 0.53 and greater than 100 µM toward RB355 cells in absence of resveratrol or with 30 or 80 µM resveratrol co-treatment. For partial dose response curves leading to a maximum of 50% inhibition or less, an IC_50_ greater than 100 µM was reported, since an accurate IC_50_ cannot be calculated.

To confirm the activity of the selected positives, eight potential agonists and 13 potential antagonists that were available from our vendors for resupply were tested in dose response in absence of resveratrol or with 30 or 80 µM resveratrol co-treatment (**Table 1**). When we compared the calculated IC_50_ values, we found that most of the resupplied potential agonists had improved potency with resveratrol co-treatment (**Fig. 4C** **,** **Table 1**), and three out of eight potential agonists had their potency improved at least two-fold in presence of 80 µM resveratrol in at least one cell line: berberine chloride, perphenazine and budesonide (**Fig. 4C** **,** **Table 1**). The most potent agonist identified, berberine chloride, was 10 fold more potent toward RB355 cells with 80 µM reveratrol co-treatment compared to no co-treatment (IC_50_ = 6.8 µM vs. 59 µM) and three fold more potent toward Y79 cells (IC_50_ = 8.6 µM vs. 24 µM) (**Fig. 5A & 5B** **,** **Table 1**). This result indicates that our screening strategy was successful, in that we have confirmed combinations with resveratrol that potentiate the effect of the selected compounds as a single agent.

**Table 1:** Summary of IC_50_ in the Alamar Blue viability assay readout for eight resupplied agonists and 13 antagonists toward the Y79 and RB355 human retinoblastoma cell lines.

			Alamar Blue viability assay IC_50_ (µM)					
			Y79			RB355		
	Compound name	Biological activity	0 µM resv.	30 µMresv.	80 µMresv.	0 µMresv.	30 µMresv.	80 µMresv.
Agonist	Berberine chloride	Antibiotic	24*	15	8.6	59*	23	6.8
	Perphenazine	Antipsychotic	32	27	16	25	19	9.8
	Avermectin B1	Antiparasitic	>100*	>100	>100	55*	47*	60*
	Phorbol 12-myristate 13 acetate	PKC activator	>100	>100	>100*	>100	>100	67*
	Budesonide	Steroid	>100	>100	>100*	>100	>100	>100*
	Piperazine carbazole ethanol derivative	Cyto C release inhibitor	9.2	7.9	7.4	12	8.7	7.0
	Triamterene	Diuretic	>100*	>100*	>100*	33*	>100*	>100*
	Lercanidipine	Ion channel inhibitor	38*	36*	38*	19	57*	33*
Antagonist	Nocodazole	Microtubule inhibitor	34*	21*	5.6*	1.1	0.53*	>100*
	Chelidonine	Microtubule inhibitor	6.8*	7.9*	4.9*	1.6	1.3*	>100*
	Albendazole	Microtubule inhibitor	>100*	>100*	>100*	20*	23*	>100*
	ZM 447439	Aurora B kinase inhibitor	25*	6.3*	4.1*	1.4	1.4	8.4
	Tolperisone	Ion channel inhibitor	74*	67*	35*	>100*	73*	49*
	PALDA	Ion channel inhibitor	5.2	13	10	6.1	15	15
	SKF 96365	Ion channel inhibitor	>100*	51*	58*	8.1	9.5	82*
	Floxuridine	Antimetabolite	0.44	0.43	0.45	0.82	0.76	1.4
	Thioguanosine	Antimetabolite	1.04	1.03	0.79	1.4	1.1	0.78
	Quinidine	NA^+^ K^+^ ATPase inhibitor	>100	>100	>100*	74*	>100	61*
	LY 171883	LTD4 antagonist	>100	>100	>100	>100	>100	>100
	Caffeic acid phenethyl ester	NFKB inhibitor	>100*	>100*	75*	21*	49*	36*
	IKK 16	IKK 16 inhibitor	3.6	4.8	6.1	5.9	6.5	6.6

Among the 13 resupplied potential antagonists, we found that five of them had their potency decreased at least two-fold in presence of 80 µM resveratrol in at least one cell line: nocodazole, chelidonine, albendazole, ZM 447439 and SKF 96365 (**Table 1**). The most potent antagonist identified, nocodazole, was less potent toward RB355 cells by more than 100-fold with 80 µM reveratrol co-treatment compared to no co-treatment (IC_50_ = 1.1 µM vs. >100 µM) (**Fig. 5C & 5D** **,** **Table 1**). Interestingly, three out of five confirmed antagonists are microtubule inhibitors: nocodazole, chelidonine and albendazole (**Table 1** **).**

**Figure 6:**
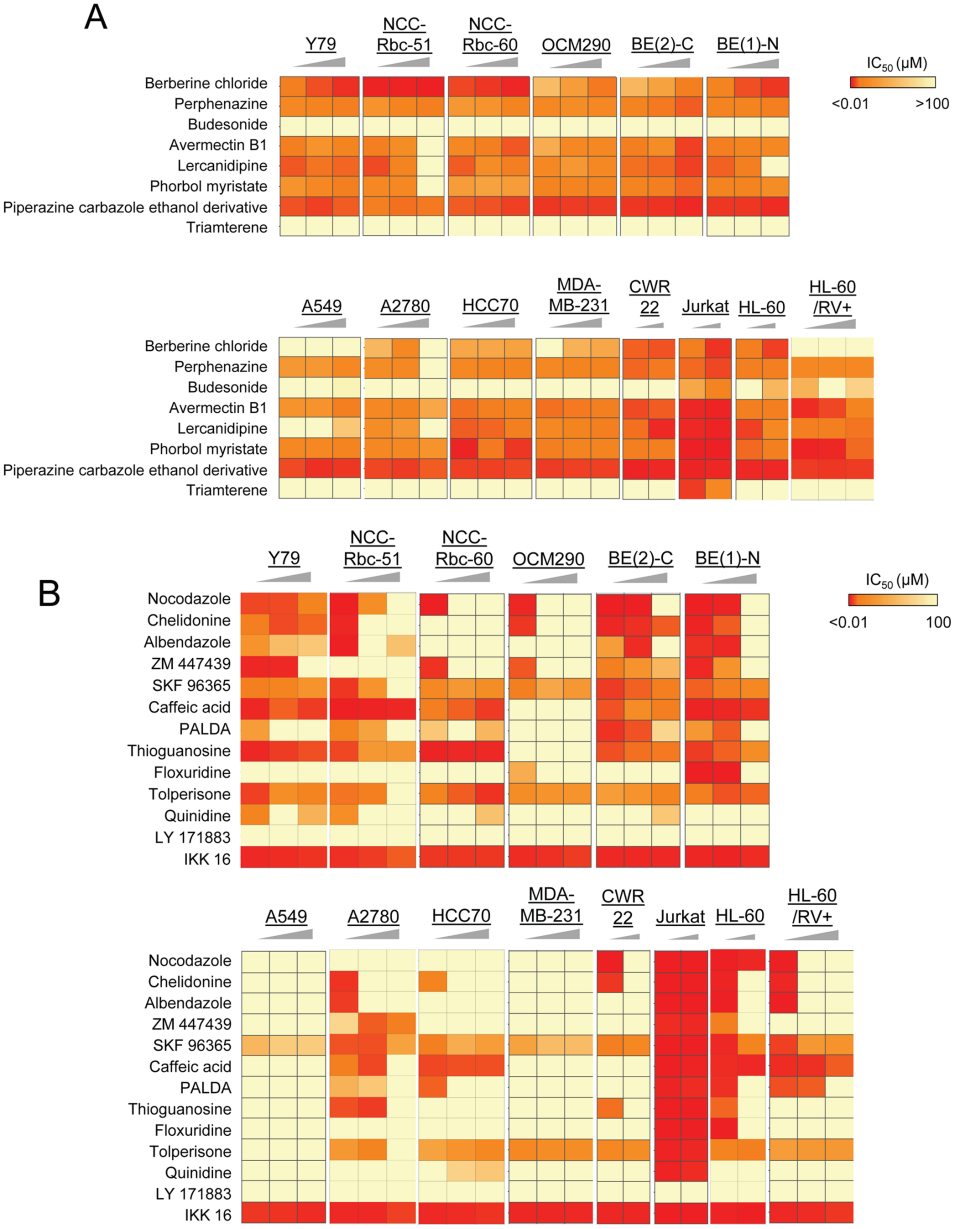
Activity profiling of confirmed positives toward a panel of cancer cell lines. Summary of potency assessment of 21 confirmed positives toward a panel of 13 cancer cell lines in absence of resveratrol or with 30 or 80 µM resveratrol co-treatment. Heat map representation of IC_50_ values in Alamar Blue-based viability assay of **A)** 8 agonists and **B)** 13 antagonists in absence of resveratrol or with 30 or 80 µM resveratrol co-treatment. The triangle symbolizes the increasing concentration in resveratrol.

**Figure 7:**
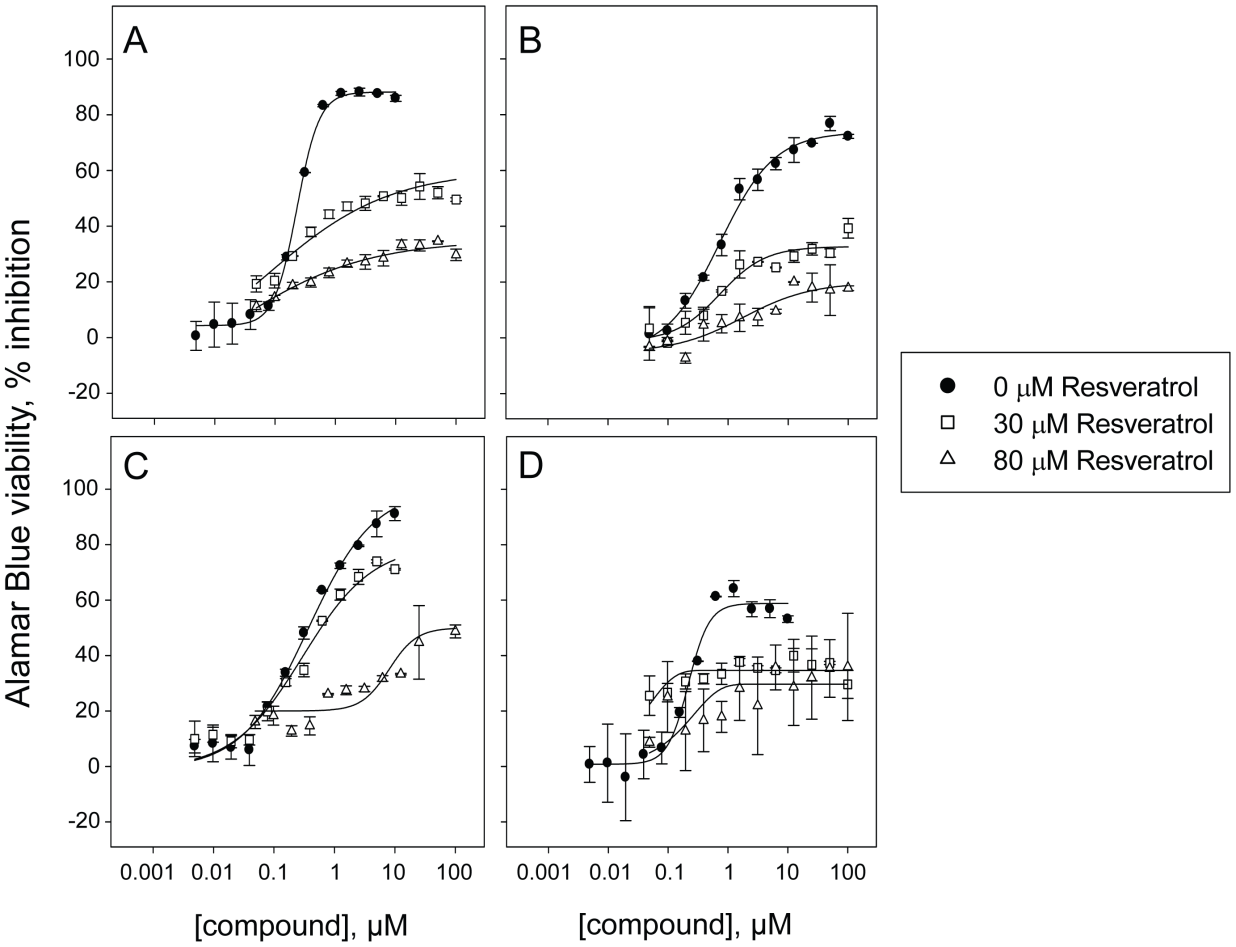
Potency assessment of the confirmed antagonist nocodazole. Dose response in absence of resveratrol or with 30 or 80 µM resveratrol co-treatment toward four cancer cell lines in an Alamar Blue-based viability assay. Dose response curves of nocodazole toward **A)** human hematopoietic cancer cell line HL-60/RV+ (IC_50_ = 0.24, greater than 100 µM and greater than 100 µM in absence of resveratrol or with 30 or 80 µM resveratrol co-treatment) **B)** human uveal melanoma cell line OCM290 (IC_50_ = 1.8, greater than 100 and greater than 100 µM in absence of resveratrol or with 30 or 80 µM resveratrol co-treatment) **C)** human neuroblastoma cell line BE(2)-C (IC_50_ = 0.35, 0.83 and greater than 100 µM in absence of resveratrol or with 30 or 80 µM resveratrol co-treatment) and **D)** human retinoblastoma cell line NCC-RbC-60 (IC_50_ = 0.23, greater than 100 µM and greater than 100 µM in absence of resveratrol or with 30 or 80 µM resveratrol co-treatment). For partial dose response curves leading to a maximum of 50% inhibition or less, an IC_50_ greater than 100 µM was reported, since an accurate IC_50_ cannot be calculated.

**Figure 8:**
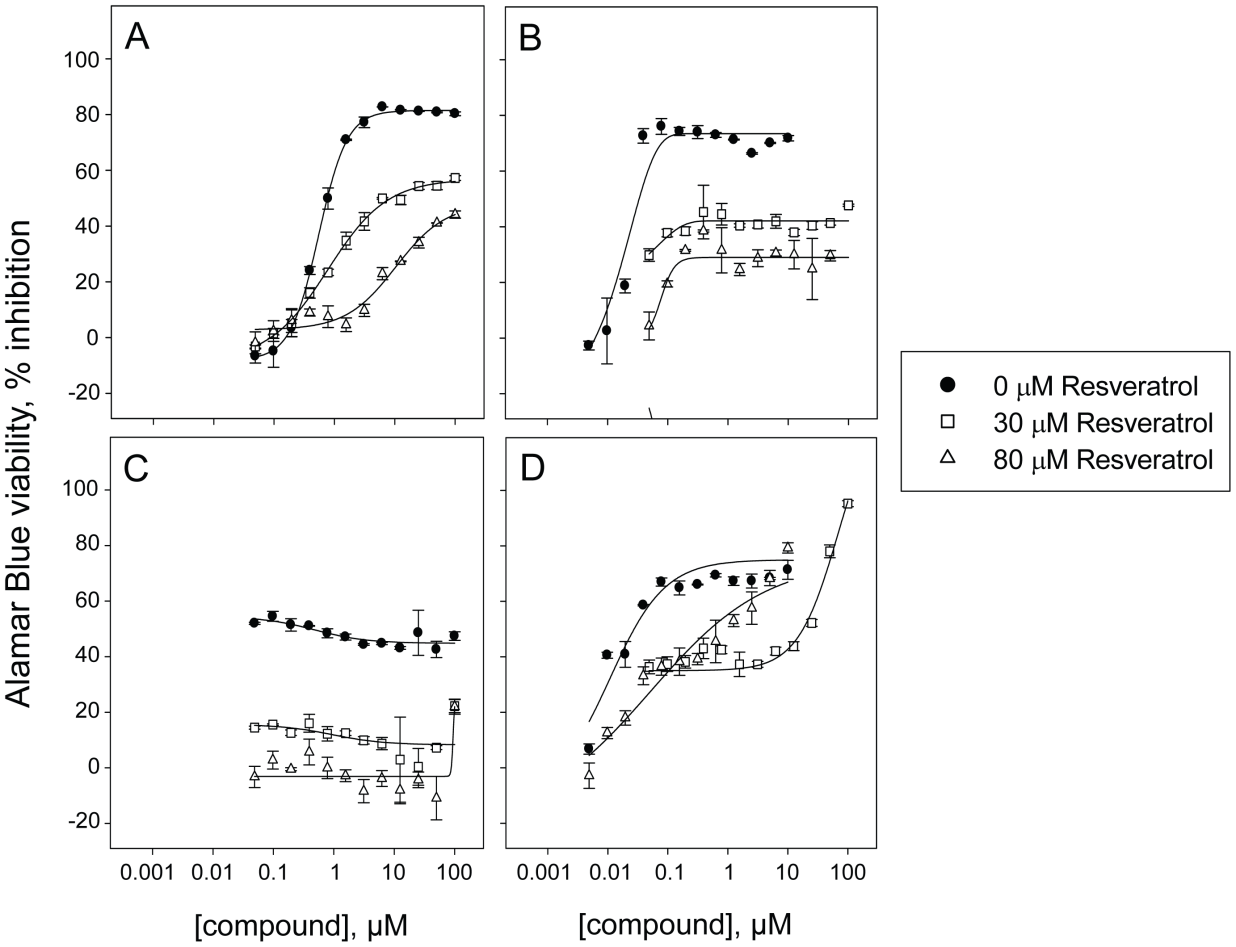
Potency assessment of the confirmed antagonist colchicine. Dose response in absence of resveratrol or with 30 or 80 µM resveratrol co-treatment toward four cancer cell lines in an Alamar Blue-based viability assay. Dose response curves of colchicine toward **A)** human hematopoietic cancer cell line HL-60/RV+ (IC_50_ = 1.06, greater than 100 and greater than 100 µM in absence of resveratrol or with 30 or 80 µM resveratrol co-treatment) **B)** human uveal melanoma cell line OCM290 (IC_50_ = 0.015, greater than 100 µM and greater than 100 µM in absence of resveratrol or with 30 or 80 µM resveratrol co-treatment) **C)** triple negative human breast cancer cell line MDA-MB-231 (IC_50_ = greater than 100 µM, greater than 100 µM and greater than 100 µM in absence of resveratrol or with 30 or 80 µM resveratrol co-treatment) and **D)** human retinoblastoma cell line NCC-RbC-60 (IC_50_ = 0.65, 3.2 and 0.74 µM in absence of resveratrol or with 30 or 80 µM resveratrol co-treatment). For partial dose response curves leading to a maximum of 50% inhibition or less, an IC_50_ greater than 100 µM was reported, since an accurate IC_50_ cannot be calculated.

**Figure 9:**
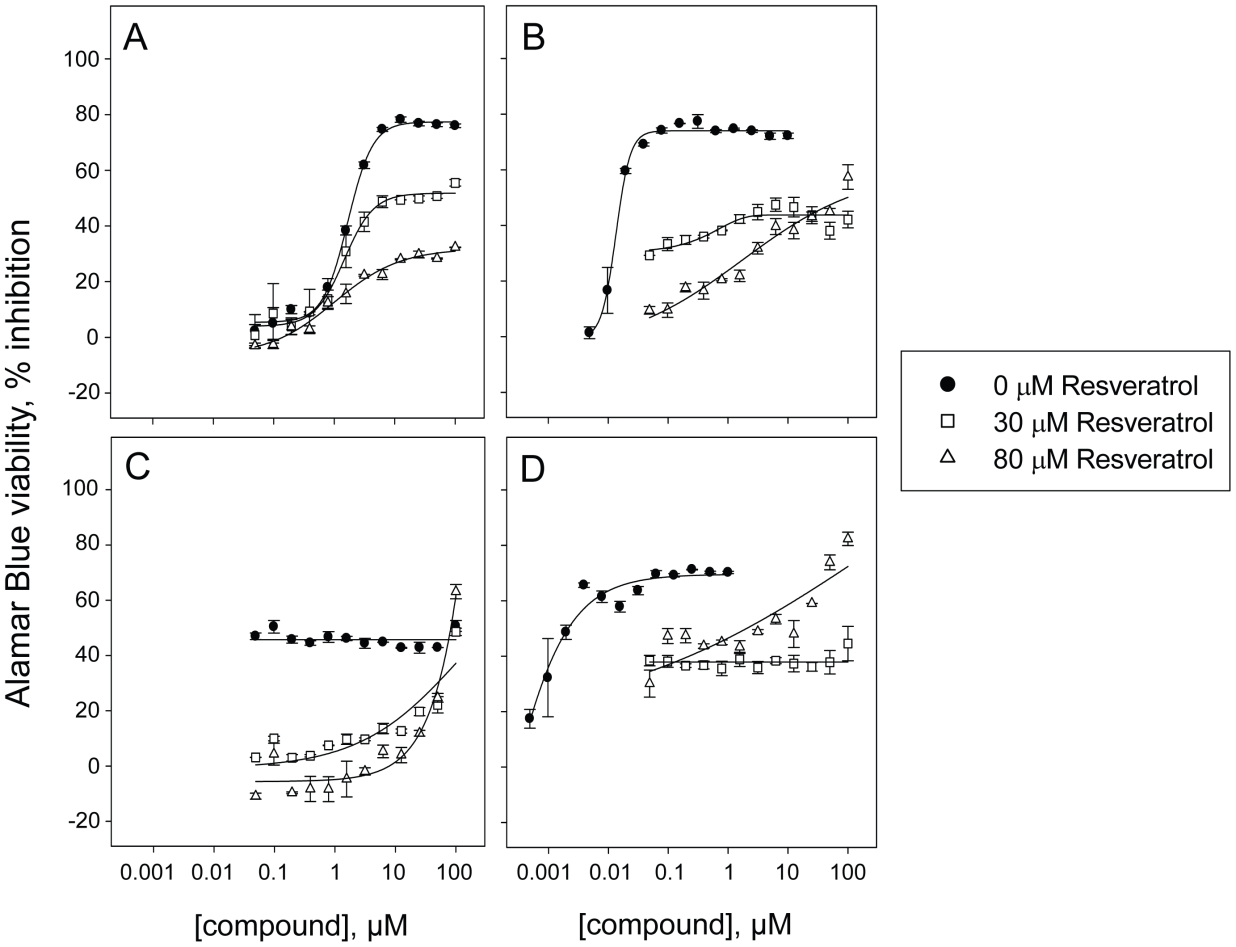
Potency assessment of the confirmed antagonist vincristine. Dose response in absence of resveratrol or with 30 or 80 µM resveratrol co-treatment toward four cancer cell lines in an Alamar Blue-based viability assay. Dose response curves of vincristine toward **A)** human hematopoietic cancer cell line HL-60/RV+ (IC_50_ = 3.1, greater than 100 µM and greater than 100 µM in absence of resveratrol or with 30 or 80 µM resveratrol co-treatment) **B)** human uveal melanoma cell line OCM290 (IC_50_ = 0.040, greater than 100 µM and greater than 100 µM in absence of resveratrol or with 30 or 80 µM resveratrol co-treatment) **C)** triple negative human breast cancer cell line MDA-MB-231 (IC_50_ = greater than 100, greater than 100 µM and greater than 100 µM in absence of resveratrol or with 30 or 80 µM resveratrol co-treatment) and **D)** human retinoblastoma cell line NCC-RbC-60 (IC_50_ = 0.010, greater than 100 µM and 1.7 µM in absence of resveratrol or with 30 or 80 µM resveratrol co-treatment). For partial dose response curves leading to a maximum of 50% inhibition or less, an IC_50_ greater than 100 µM was reported, since an accurate IC_50_ cannot be calculated.

To establish whether our findings are limited to retinoblastoma cells or whether they have a broader scope, we screened in dose response all resupplied agonists and antagonists in a panel of 13 human cell lines covering a broad range of cancer types. Based on our findings, we included in this study two additional microtubule inhibitors: vincristine and colchicine. Among the eight resupplied potential agonists, we found that not only the three agonists confirmed with retinoblastoma cells were also agonists in other cell lines, but two additional compounds were as well: avermectin B1 and lercadinipine (**Fig. 6A** **).** In total, five out eight potential agonists selected from screening were at least twice more potent in presence of 30 or 80 µM resveratrol, and toward at least three non-retinoblastoma cell lines (**Table S1).** Among the 13 resupplied potential antagonists, we found that not only the five antagonists confirmed with retinoblastoma cells were also potent toward other cell lines, but four additional compounds were as well: caffeic acid, PALDA, thioguanosine and floxuridine (**Fig. 6B** **).** In total, nine out of thirteen potential antagonists selected from screening were at least twice more potent in presence of 30 or 80 µM resveratrol and toward at least three non-retinoblastoma cell lines (**Table S1).** Importantly, all microtubule inhibitors included in this study were confirmed as antagonists across at least three non-retinoblastoma cell lines with shifts in potency greater than 400 fold for nocodazole (**Fig. 7** **,** **Table S1)** and even up to greater than 2,500- or 6,000-fold toward OCM290 cells, for vincristine and colchicine respectively **(** **Fig. 8** **,** **Fig. 9** **and Table S1).**

So far in this study, our assessment of compound-induced antiproliferative effect was based on an Alamar Blue-based viability readout. To further characterize the activity of identified agonists and antagonists, we evaluated their effect on cell proliferation as measured by automated image-based direct nuclei count using the same dose response plates used for the Alamar Blue readout. Of note, we could perform this readout only for adherent cell lines, and we found that we could reliably calculate IC_50_ values for all screening conditions (0, 30 and 80 µM resveratrol) only for the uveal melanoma cell line OCM290. This was expected, as the cell seeding densities were optimized for Alamar Blue readout and not for nuclei count. Importantly, we found that all but three antagonists identified in the screen (PALDA, quinidine and LY 171883) had their potency significantly shifted toward being less potent as measured using the direct nuclei count readout of cell proliferation (**Table 2**). The observed antagonistic effect followed the same trend as measured in the viability readout, but was more pronounced in the cell proliferation readout: microtubule inhibitors such as colchicine and vincristine were found to lose their potency by up to greater than 5,000- to 10,000-fold. Besides microtubule inhibitors, potent antagonists in this readout were the antimetabolite floxuridine (>625-fold) and the alkaloid chelidonine (>50-fold). Of note, agonists in the viability readout were not found to constitute agonists in the cell proliferation readout (**Table 2**). This result suggests that the the identified agonists affect the metabolism of treated cells, but not their proliferation, at least not in a way that could be measured during the 120 h timeframe of this assay.

**Table 2:** Summary of IC_50_ in the nuclei count readout for eight resupplied agonists, 13 antagonists and two additional compounds toward the human uveal melanoma cell line OCM290.

			Nuclei Count Proliferation assay IC_50_ (µM)		
	Compound name	Class	0 µM resv.	30 µM resv.	80 µM resv.
Agonist	Berberine chloride	Antibiotic	10*	>100*	>100*
	Perphenazine	Antipsychotic	19*	38*	26*
	Avermectin B1	Antiparasitic	6.9*	>100*	19*
	Phorbol 12-myristate 13 acetate	PKC activator	11*	21*	17*
	Budesonide	Steroid	>100	>100	>100
	Piperazine carbazole ethanol derivative	Cyto C release inhibitor	3.8	5.2*	6.2*
	Triamterene	Diuretic	27*	>100	>100
	Lercanidipine	Ion channel inhibitor	8.7	>100*	>100*
Antagonist	Nocodazole	Microtubule inhibitor	0.19*	>100*	>100
	Chelidonine	Microtubule inhibitor	2.0*	>100*	>100
	Albendazole	Microtubule inhibitor	0.93	>100*	>100*
	ZM 447439	Aurora B kinase inhibitor	0.55	>100*	>100*
	Tolperisone	Ion channel inhibitor	14	34*	58*
	PALDA	Ion channel inhibitor	>100*	>100	>100
	SKF 96365	Ion channel inhibitor	2.7	6.0*	37*
	Floxuridine	Antimetabolite	0.16	>100	>100
	Thioguanosine	Antimetabolite	7.1	>100*	>100*
	Quinidine	NA^+^ K^+^ ATPase inhibitor	>100*	>100	>100*
	LY 171883	LTD4 antagonist	>100	>100	>100
	Caffeic acid phenethyl ester	NFKB inhibitor	18*	>100*	>100
	IKK 16	IKK 16 inhibitor	1.3	4.4	4.6
Additional compounds	Vincristine sulfate	Microtubule inhibitor	0.010*	>100*	>100*
	Colchicine	Microtubule inhibitor	0.020	>100*	>100*

**Figure 10:**
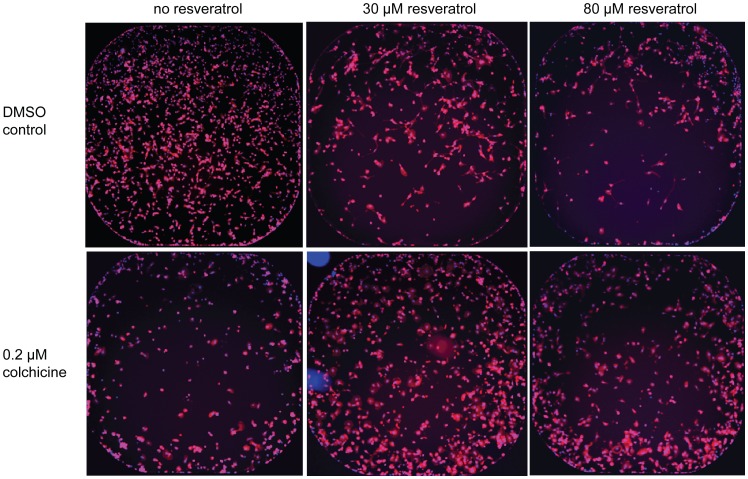
4X images of colchicine treated MDA-MB231. INCA 2000 images of triple negative human breast cancer cells MDA-MB-231 at 4X objective magnification showing Hoechst-stained nuclei (blue) and rhodamine phalloidin-stained actin (red) for high control 1% DMSO and 0.2 µM colchicine in absence of resveratrol or with 30 or 80 µM resveratrol co-treatment.

**Figure 11:**
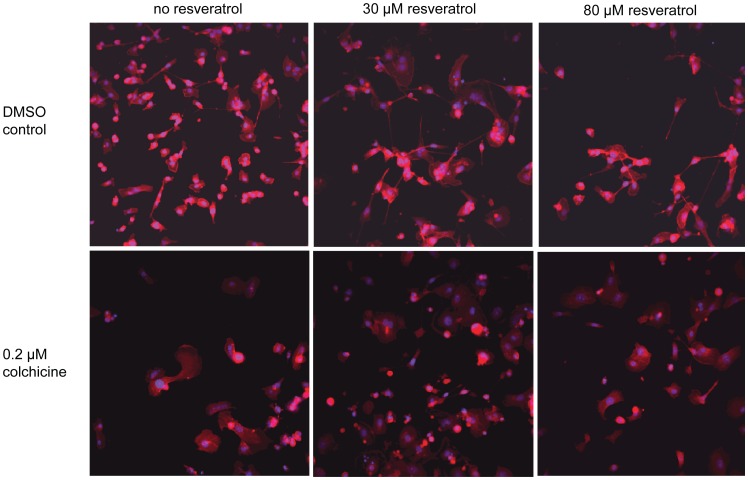
20X images of colchicine treated MDA-MB231. INCA 2000 images of triple negative human breast cancer cells MDA-MB-231 at 20X objective magnification showing Hoechst-stained nuclei (blue) and rhodamine phalloidin-stained actin (red) for high control 1% DMSO and 12.5 µM colchicine in absence of resveratrol or with 30 or 80 µM resveratrol co-treatment.

**Figure 12:**
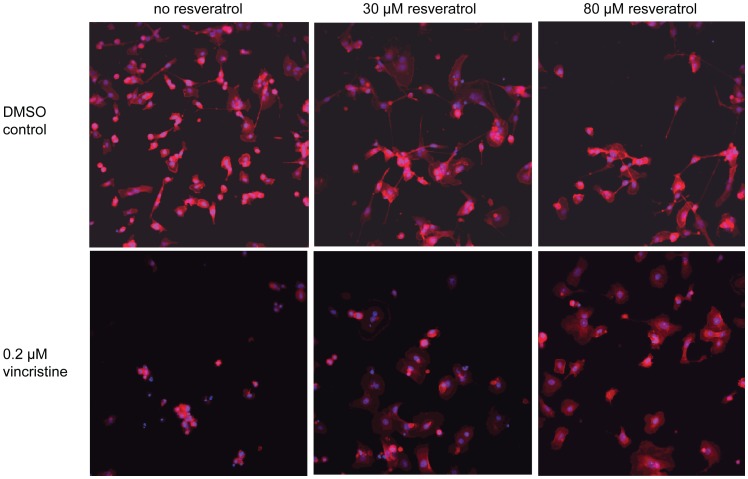
20X images of vincristine treated MDA-MB231. INCA 2000 images of triple negative human breast cancer cells MDA-MB-231 at 20X objective magnification showing Hoechst-stained nuclei (blue) and rhodamine phalloidin-stained actin (red) for high control 1% DMSO and 0.2 µM vincristine in absence of resveratrol or with 30 or 80 µM resveratrol co-treatment.

To confirm our observations and to characterize the morphology of cells treated with antagonists, we imaged cells from the same plates used for the viability and proliferation readout following actin staining. Whole well images of human triple negative breast cancer cells MDA-MB-231 treated with 0.2 µM colchicine illustrate the antiproliferative effect of this microtubule inhibitor after 120 h incubation compared to DMSO control (**Fig. 10**). As expected, treatment of MDA-MB-231 cells with 30 and 80 µM resveratrol induces a dose-dependent decrease in proliferation, but remarkably, co-treatment of these cells with colchicine and resveratrol drastically reverses the antiproliferative effect of both drugs (**Fig. 10**). Higher magnification images reveal that co-treated cells seem to have an enlarged cytoplasm (**Fig. 11**). We made again the same observation when cells were co-treated with vincristine and resveratrol (**Fig. 12**). These observations confirm our results, in that co-treatment of cancer cells with microtubule inhibitors and resveratrol results in the antagonization of the antiproliferative effect of the microtubule inhibitor. A broader implication of this finding is the confirmation that our platform can identify potent combinatorial effects with approved drugs induced by a companion effector such as resveratrol.

## Discussion

Combining drugs acting on multiple targets is increasingly seen as a necessity to achieve sustained tumor regression in patients by increasing potency and decreasing resistance to treatment [2], [3], [5]. However, the current approach consisting of testing combinations in patients is slow and expensive [5] and as of today, while an initiative derived from the NCI60 anticancer screen is under way [7], current preclinical studies of drug combinations are often biased toward certain targets [5] and may very well yield increased toxicity in patients due to the combination of off-target effects [8].

As a different approach, we sought to take advantage of a companion effector known to be safe in patients such as a nutraceutical, offering potential therapeutic benefits but limited by low potency. To validate our strategy, we aimed at identifying compounds that would synergize with the antioxidant resveratrol widely used as a supplement to yield antiproliferative effect toward retinoblastoma cells. Resveratrol, a natural product with antioxidant properties is among the most commercially successful nutraceuticals, due to putative health benefits such as anti-inflammatory, anti-aging properties and prevention of cardiovascular diseases, diabetes and cancer [9], [19], supported by more than 4,000 scientific publications [9]. Rapidly absorbed after oral consumption, resveratrol is also rapidly metabolized, leading to low circulating concentration in patients that limits its use as a single agent due to combined low potency [20].

We established a platform that enables the rapid in vitro identification of synergetic pairs between resveratrol as a companion effector and approved drugs, and comparative analysis of screening of over 6,000 bioactive compounds and FDA-approved drugs performed in two retinoblastoma cell models and in presence or absence of resveratrol co-treatment revealed agonists and antagonists (**Fig. 4**), confirmed in dose response among a panel of 13 cancer cell lines (**Fig. 6**). This is an important result, since it validates our alternative screening approach.

A few potential agonists were identified in this study, such as berberine chloride, but while the effect of many of them was confirmed in dose response across a panel of cell lines in the viability readout (**Fig. 5** **&** **6** **, Table S1**), none of them were found to have a significant agonistic effect in the direct nuclei count-based proliferation readout (**Table 2**). This observation indicates that the identified agonists may affect cell metabolism, but not their proliferation during the timeframe of this assay. Further studies are needed to evaluate the potential of the additive combinations we identified.

To our surprise, we discovered an antagonism between microtubule inhibitors commonly used in the clinic as anticancer agents and resveratrol. We confirmed this observation across a panel of cancer cell lines using both viability and proliferation readouts, and with four different compounds interfering with microtubules: the chemically-related drugs albendazole and nocodazole identified during the screen, as well as the structurally distinct drugs vincristine and colchicine, (**Fig. 8** **,** **9** **,** **10** **,** **11** **,** **12**, **Table 1** **and** **2** **, Table S1**). Interestingly, resveratrol was previously found to inhibit apoptosis induced by vincristine [30] and to block the cytotoxic effect of the antimicrotubule agent vinblastine and of the microtubule stabilizer taxol [31]. The mechanism of action is unclear, consistent with multiple known molecular targets for resveratrol [32], as in the first study the authors report that blocking the activation of NADPH oxidase neutralized apoptosis induced by resveratrol [30], while the second study concludes that resveratrol prevents taxol to exert its action by inducing cell cycle arrest [31]. Our study expands the scope of this observation to four agents interfering with microtubules, and across multiple cell lines covering a broad range of cancer types. In addition, among the confirmed antagonists was the Aurora B kinase inhibitor ZM447439, which is known to interfere with mitotic spindle assembly by inhibiting the formation of microtubules [33]. Altogether, our results consistent with previous findings suggest a link between resveratrol and microtubule assembly, but further studies are needed to investigate the mechanism responsible for this antagonistic effect. While our observations in vitro may not apply to patients, for example due to lack of drug bioavailability, their potential clinical implication is of importance, as the commercial success of resveratrol as a nutraceutical is tremendous and cancer patients are tempted to consume resveratrol supplements to improve their health, which could potentially interfere with their treatment if it consists of a microtubule inhibitor. Whether our in vitro observation would translate into patients remains to be further evaluated, since repeated administration of resveratrol yields relatively low plasma concentrations (in the range of 2 µM as the peak concentration), even at high dose and short intervals [19]. Regardless of any validation in patients, our observations further support the use of caution with resveratrol supplements, as resveratrol is already known to inhibit platelet aggregation [34], potentially inducing additive effects with anticoagulant drugs, and may also interfere with the metabolism of other drugs by inhibiting cytochrome expression or activity [35], [36]. Our findings illustrate the potential of our approach for uncovering potential drug interactions with nutraceuticals.

The current method to identify synergetic combinations consists of rationally designing combinations of approved drugs predicted to have advantages compared to the administration of each drug as a single agent, and to test this hypothesis in a dedicated clinical trial. This approach is long and expensive, and arguably conceptually flawed, since such a rational design of combinations is limited by our lack of knowledge of the complex signaling networks governing cell processes and the unknown in-depth specificity profile of current or prospective drugs such as kinase inhibitors [3], [5]. In addition, rational design is inadequate for compounds with an unknown mechanism of action such as resveratrol, and with the renewed emphasis on phenotypic screens, more and more drugs will be identified in absence of any knowledge of their molecular target. Our in vitro platform addresses those limitations, as demonstrated in this article. First, our proven workflow allows the rapid identification and characterization of combinations of interest by screening chemical libraries at one concentration in presence or absence of bait compound. Activity profiling in dose response in a broad panel of cell lines confirmed the activity of most identified agonists and antagonists. Second, our unbiased approach allows target serendipity: the discovery of combinatorial effects that could not have been predicted, as demonstrated by the antagonism between microtubule inhibitors and resveratrol.

In summary, this article recapitulates our efforts in establishing a novel platform for combination screening. We devised an alternative strategy for the identification of synergetic pairs with approved drugs, seeking to take advantage of nutraceuticals consumed on a daily basis by a large population and known to be safe. Our proof of concept screen with resveratrol as companion effector validates our approach: though we did not identify actual synergizers, we stumbled upon a class of anticancer drugs widely used in the clinic whose antiproliferative effect is antagonized when combined with resveratrol. This result, if confirmed in vivo, could potentially explain in part why some patients do not respond to treatment, highlighting how our approach could significantly impact both drug discovery and the nutraceutical industry.

## Acknowledgments

The authors would like to thank all members of the HTS Core Facility for their help during the course of this study.

## Supporting Information

Table S1 **Summary of IC_50_ values across the panel of 14 cell lines.**
(XLSX)

## References

[1] DeVita VT Jr, Rosenberg SA (2012) Two hundred years of cancer research. N Engl J Med 366: 2207–2214. 22646510 10.1056/NEJMra1204479 PMC6293471

[2] Bock C, Lengauer T (2012) Managing drug resistance in cancer: lessons from HIV therapy. Nat Rev Cancer 12: 494–501. 22673150 10.1038/nrc3297

[3] Kummar S, Chen HX, Wright J, Holbeck S, Millin MD, et al (2010) Utilizing targeted cancer therapeutic agents in combination: novel approaches and urgent requirements. Nat Rev Drug Discov 9: 843–856. 21031001 10.1038/nrd3216

[4] Gorre ME, Mohammed M, Ellwood K, Hsu N, Paquette R, et al (2001) Clinical resistance to STI-571 cancer therapy caused by BCR-ABL gene mutation or amplification. Science 293: 876–880. 11423618 10.1126/science.1062538

[5] Knight ZA, Lin H, Shokat KM (2010) Targeting the cancer kinome through polypharmacology. Nat Rev Cancer 10: 130–137. 20094047 10.1038/nrc2787 PMC2880454

[6] Chapman PB, Hauschild A, Robert C, Haanen JB, Ascierto P, et al (2011) Improved survival with vemurafenib in melanoma with BRAF V600E mutation. N Engl J Med 364: 2507–2516. 21639808 10.1056/NEJMoa1103782 PMC3549296

[7] Holbeck S, Collins J, Doroshow J (2012) 27 NCI-60 Combination Screening Matrix of Approved Anticancer Drugs. European Journal of Cancer 48: 11.

[8] Chen MH, Kerkela R, Force T (2008) Mechanisms of cardiac dysfunction associated with tyrosine kinase inhibitor cancer therapeutics. Circulation 118: 84–95. 18591451 10.1161/CIRCULATIONAHA.108.776831 PMC2735334

[9] Rossi D, Guerrini A, Bruni R, Brognara E, Borgatti M, et al (2012) trans-Resveratrol in Nutraceuticals: Issues in Retail Quality and Effectiveness. Molecules 17: 12393–12405. 23090020 10.3390/molecules171012393 PMC6268383

[10] Subramanian L, Youssef S, Bhattacharya S, Kenealey J, Polans AS, et al (2010) Resveratrol: challenges in translation to the clinic–a critical discussion. Clin Cancer Res 16: 5942–5948. 21045084 10.1158/1078-0432.CCR-10-1486 PMC3057445

[11] Li JW, Vederas JC (2009) Drug discovery and natural products: end of an era or an endless frontier? Science 325: 161–165. 19589993 10.1126/science.1168243

[12] Qin C, Tan KL, Zhang CL, Tan CY, Chen YZ, et al (2012) What Does It Take to Synergistically Combine Sub-Potent Natural Products into Drug-Level Potent Combinations? PLoS One 7: e49969. 23209627 10.1371/journal.pone.0049969 PMC3509152

[13] Chantada GL, Qaddoumi I, Canturk S, Khetan V, Ma Z, et al (2011) Strategies to manage retinoblastoma in developing countries. Pediatr Blood Cancer 56: 341–348. 21225909 10.1002/pbc.22843

[14] Antczak C, Kloepping C, Radu C, Genski T, Muller-Kuhrt L, et al (2009) Revisiting old drugs as novel agents for retinoblastoma: in vitro and in vivo antitumor activity of cardenolides. Invest Ophthalmol Vis Sci 50: 3065–3073. 19151399 10.1167/iovs.08-3158 PMC3617409

[15] Patel M, Paulus YM, Gobin YP, Djaballah H, Marr B, et al (2011) Intra-arterial and oral digoxin therapy for retinoblastoma. Ophthalmic Genet 32: 147–150. 21446853 10.3109/13816810.2010.544530

[16] Sareen D, van Ginkel PR, Takach JC, Mohiuddin A, Darjatmoko SR, et al (2006) Mitochondria as the primary target of resveratrol-induced apoptosis in human retinoblastoma cells. Invest Ophthalmol Vis Sci 47: 3708–3716. 16936077 10.1167/iovs.06-0119

[17] van Ginkel PR, Darjatmoko SR, Sareen D, Subramanian L, Bhattacharya S, et al (2008) Resveratrol inhibits uveal melanoma tumor growth via early mitochondrial dysfunction. Invest Ophthalmol Vis Sci 49: 1299–1306. 18385041 10.1167/iovs.07-1233 PMC2465765

[18] Fulda S (2010) Resveratrol and derivatives for the prevention and treatment of cancer. Drug Discov Today 15: 757–765. 20692359 10.1016/j.drudis.2010.07.005

[19] Vang O, Ahmad N, Baile CA, Baur JA, Brown K, et al (2011) What is new for an old molecule? Systematic review and recommendations on the use of resveratrol. PLoS One 6: e19881. 21698226 10.1371/journal.pone.0019881 PMC3116821

[20] Scott E, Steward WP, Gescher AJ, Brown K (2012) Resveratrol in human cancer chemoprevention–choosing the ‘right’ dose. Mol Nutr Food Res 56: 7–13. 22218912 10.1002/mnfr.201100400

[21] Soto BL, Hank JA, Van De Voort TJ, Subramanian L, Polans AS, et al (2011) The anti-tumor effect of resveratrol alone or in combination with immunotherapy in a neuroblastoma model. Cancer Immunol Immunother 60: 731–738. 21340652 10.1007/s00262-011-0971-0 PMC3094716

[22] Fournier GA, Sang DN, Albert DM, Craft JL (1987) Electron microscopy and HLA expression of a new cell line of retinoblastoma. Invest Ophthalmol Vis Sci 28: 690–699. 3549617

[23] Hu J, Cheung NK (2009) Methionine depletion with recombinant methioninase: in vitro and in vivo efficacy against neuroblastoma and its synergism with chemotherapeutic drugs. Int J Cancer 124: 1700–1706. 19089915 10.1002/ijc.24104 PMC2700741

[24] Ambrosini G, Schwartz G (2010) The MEK inhibitor AZD6244 is active in GNAQ mutant ocular melanoma cells. 17–21.

[25] Ma LD, Marquardt D, Takemoto L (1991) Center MS (1991) Analysis of P-glycoprotein phosphorylation in HL60 cells isolated for resistance to vincristine. J Biol Chem 266: 5593–5599. 1672314

[26] Antczak C, Mahida JP, Bhinder B, Calder PA, Djaballah H (2012) A high-content biosensor-based screen identifies cell-permeable activators and inhibitors of EGFR function: implications in drug discovery. J Biomol Screen 17: 885–899. 22573732 10.1177/1087057112446174 PMC3615554

[27] Lee G, Ramirez CN, Kim H, Zeltner N, Liu B, et al.. (2012) Large-scale screening using familial dysautonomia induced pluripotent stem cells identifies compounds that rescue IKBKAP expression. Nat Biotechnol. 10.1038/nbt.2435 PMC3711177 23159879

[28] Shum D, Radu C, Kim E, Cajuste M, Shao Y, et al (2008) A high density assay format for the detection of novel cytotoxic agents in large chemical libraries. J Enzyme Inhib Med Chem 23: 931–945. 18608772 10.1080/14756360701810082 PMC3710589

[29] Antczak C, Shum D, Escobar S, Bassit B, Kim E, et al (2007) High-throughput identification of inhibitors of human mitochondrial peptide deformylase. J Biomol Screen 12: 521–535. 17435169 10.1177/1087057107300463 PMC2234356

[30] Ahmad KA, Clement MV, Hanif IM, Pervaiz S (2004) Resveratrol inhibits drug-induced apoptosis in human leukemia cells by creating an intracellular milieu nonpermissive for death execution. Cancer Res 64: 1452–1459. 14973069 10.1158/0008-5472.can-03-2414

[31] Mao QQ, Bai Y, Lin YW, Zheng XY, Qin J, et al (2010) Resveratrol confers resistance against taxol via induction of cell cycle arrest in human cancer cell lines. Mol Nutr Food Res 54: 1574–1584. 20521268 10.1002/mnfr.200900392

[32] Athar M, Back JH, Kopelovich L, Bickers DR, Kim AL (2009) Multiple molecular targets of resveratrol: Anti-carcinogenic mechanisms. Arch Biochem Biophys 486: 95–102. 19514131 10.1016/j.abb.2009.01.018 PMC2749321

[33] Gadea BB, Ruderman JV (2005) Aurora kinase inhibitor ZM447439 blocks chromosome-induced spindle assembly, the completion of chromosome condensation, and the establishment of the spindle integrity checkpoint in Xenopus egg extracts. Mol Biol Cell 16: 1305–1318. 15616188 10.1091/mbc.E04-10-0891 PMC551494

[34] Pace-Asciak CR, Hahn S, Diamandis EP, Soleas G, Goldberg DM (1995) The red wine phenolics trans-resveratrol and quercetin block human platelet aggregation and eicosanoid synthesis: implications for protection against coronary heart disease. Clin Chim Acta 235: 207–219. 7554275 10.1016/0009-8981(95)06045-1

[35] Chen ZH, Hurh YJ, Na HK, Kim JH, Chun YJ, et al (2004) Resveratrol inhibits TCDD-induced expression of CYP1A1 and CYP1B1 and catechol estrogen-mediated oxidative DNA damage in cultured human mammary epithelial cells. Carcinogenesis 25: 2005–2013. 15142886 10.1093/carcin/bgh183

[36] Chun YJ, Kim MY, Guengerich FP (1999) Resveratrol is a selective human cytochrome P450 1A1 inhibitor. Biochem Biophys Res Commun 262: 20–24. 10448061 10.1006/bbrc.1999.1152

